# A Single-Cell Perspective on Remapping Human Adult Neurogenesis and Its Clinical Implications

**DOI:** 10.3390/biom16020331

**Published:** 2026-02-22

**Authors:** Xin Tian, Renqing Zhao

**Affiliations:** College of Physical Education, Yangzhou University, Yangzhou 225009, China; tianxin96331@163.com

**Keywords:** hippocampal neurogenesis, single-cell RNA sequencing, neurological diseases

## Abstract

Recent advances in single-cell RNA sequencing (scRNA-seq) have substantially deepened our understanding of adult hippocampal neurogenesis (AHN), enabling the detection of neural stem cells, progenitors, and immature neurons in postmortem *human* brain tissue and revealing how these populations are altered in neurological disease. Additionally, scRNA-seq enables the identification of disease-specific cell subtypes and distinct gene expression signatures associated with neurological disorders, many of which are linked to alterations in AHN and cognitive function. Such cellular- and molecular-level insights into neurological disease mechanisms provide a strong foundation for the development of targeted therapeutic strategies. Indeed, scRNA-seq has also emerged as a powerful tool in drug discovery and development across multiple disease areas, including cancer, cardiovascular disorders, and neurological conditions. In this review, we offer a comprehensive and integrative perspective on the cellular and molecular landscape of *human* hippocampal neurogenesis, the pathological mechanisms underlying neurological disorders, and their implications for therapeutic development.

## 1. Introduction

Despite significant strides in neuroscience over recent decades, numerous fundamental enigmas persist, necessitating innovative methodological frameworks for their resolution [1,2,3]. A paramount challenge lies in deciphering the functional significance and regulatory architecture of adult hippocampal neurogenesis (AHN) in the *human* brain [4,5,6]. Conventional techniques, such as immunohistochemistry (IHC), face considerable challenges in the study of *human* AHN, including the presumed scarcity of newly generated neurons, the vulnerability of postmortem brain tissue, and the absence of consistently reliable markers for adult-born neurons [7,8,9]. Key markers, notably doublecortin (DCX), are especially prone to degradation during tissue processing, which may contribute to conflicting observations across studies. In parallel, bulk RNA sequencing yields only averaged regional profiles and is inherently unable to capture critical cell-type-specific and single-cell transcriptomic heterogeneity, rare populations, and dynamic cell-state transitions [10,11,12]. The advent of single-cell RNA sequencing (scRNA-seq) has begun to overcome these barriers [13,14,15,16], providing a high-resolution window into the complexities of *human* hippocampal neurogenesis [1,17], its governing mechanisms, and prospective therapeutic avenues [18].

Neural stem cells (NSCs) are self-renewing, multipotent cells residing in specialized neurogenic niches, such as the subgranular zone of the dentate gyrus (DG) and the subventricular zone, where they generate neurons, astrocytes, and oligodendrocytes, thereby contributing to neuronal plasticity [19]. While AHN is established as a conserved adaptive mechanism in rodents and non-*human* primates [20,21,22,23], its existence and magnitude in the adult *human* hippocampus remain a subject of intense debate [5,24,25]. The emerging technique of scRNA-seq, which enables large-scale transcriptomic profiling at single-cell resolution, has provided deep insight into the molecular complexity of brain cell populations [1,23,26]. This approach has been widely used to identify cell subtypes, reconstruct lineage trajectories, and uncover genetic determinants of adult neurogenesis in brain tissue [1,23,26]. Accumulating studies highlight the utility of scRNA-seq in resolving transcriptomic profiles at the level of individual cells or nuclei from brain tissue, as well as in clarifying the involvement of AHN in neurodegeneration-associated brain dysfunction [27,28,29]. Accordingly, a systematic understanding of how scRNA-seq has shaped AHN research—and how it can be leveraged to dissect the mechanisms of neurological disease—will be critical for advancing the development of targeted therapeutic strategies.

scRNA-seq is increasingly recognized as a groundbreaking tool in the investigation and management of neurological diseases [13,14,15,16]. Its integration into clinical research and practice holds considerable promise for improving diagnostic accuracy, prognostic assessment, and therapeutic intervention. By delineating disease-specific transcriptional signatures, scRNA-seq provides a robust framework for refined disease classification and the identification of previously unrecognized cellular subpopulations. Moreover, interrogation of gene expression at single-cell resolution enables detailed dissection of disease-associated mechanisms and signaling pathways. Through the comparative analysis of transcriptomic profiles between healthy and pathological tissues, researchers can discern disease-specific gene signatures and decode intricate regulatory networks. While significant advancements have been made in utilizing scRNA-seq to unveil novel therapeutic targets, many questions remain to be addressed, such as validating specific cellular and molecular alterations in neurological disorders and confirming their therapeutic efficacy in diverse disease models. An active area of investigation, for instance, focuses on elucidating potential links between variability in AHN and pathological burdens such as tau and α-synuclein accumulation, with the ultimate goal of improving AHN and cognitive function. Single-cell–level analyses of gene expression phenotypes using scRNA-seq facilitate the discovery of disease-associated signatures and regulatory signaling pathways that may represent candidate therapeutic targets. Therefore, this review synthesizes critical inquiries and emerging evidence regarding the application of scRNA-seq in *human* adult neurogenesis and its intersection with neurological pathology. Specifically, we address the following key domains: (i) the progress of scRNA-seq analysis in revealing *human* AHN; (ii) the relationship between AHN and neurological disorders from a single-cell perspective; (iii) understanding the cellular and molecular mechanisms of neurological disorders; and (iv) the challenges and future directions.

## 2. scRNA-Seq Brings New Dimensions to Neurology Research

Recent years have witnessed a surge in research focused on *human* AHN, propelled by emerging snRNA-seq evidence that offers fresh insights into its existence, functional roles, and governing regulatory frameworks [30,31]. As a potent analytical advancement, scRNA-seq enables the interrogation of transcriptomic profiles at the individual-cell level, yielding a high-resolution atlas of cellular states—a capability of paramount importance for dissecting the AHN landscape [32,33,34]. In contrast to conventional techniques such as IHC, which provide limited information from postmortem brain tissue, scRNA-seq allows the identification of rare cell populations, reveals heterogeneity within ostensibly homogeneous cell groups, traces neural progenitor lineage trajectories, and uncovers gene regulatory networks governing cell fate decisions [27,28,29]. In neurological disease contexts, resolving these processes at single-cell resolution offers valuable opportunities for identifying potential therapeutic targets [35,36]. For instance, factors that support the survival and functional integration of newly generated neurons in the hippocampus may be leveraged to enhance AHN and cognitive function—processes that are frequently compromised in disorders such as Alzheimer’s disease (AD), Parkinson’s disease (PD), and depression [37,38]. Moreover, rapid advances in single-cell technologies have provided new insights into cell-specific drug responses, potential off-target effects, and intercellular variability, thereby facilitating the selection of the most promising therapeutic candidates [39,40,41,42]. These technologies enhance our understanding of neurological research and practice by identifying biomarkers that aid in patient stratification, clarifying the mechanisms underlying a drug’s effects or resistance patterns, and tracking the progression of responses to both the drug and the disease [18]. This facilitates a more personalized approach to treatment, potentially leading to more effective and targeted therapies. Collectively, the rapid development of scRNA-seq methodologies provides a promising opportunity for neuroscience research, bringing novel dimensions to understanding and treating brain disorders.

## 3. scRNA-Seq Advancing the Study of AHN in Mammals

### 3.1. Molecular Regulation of AHN

**Figure 1:**
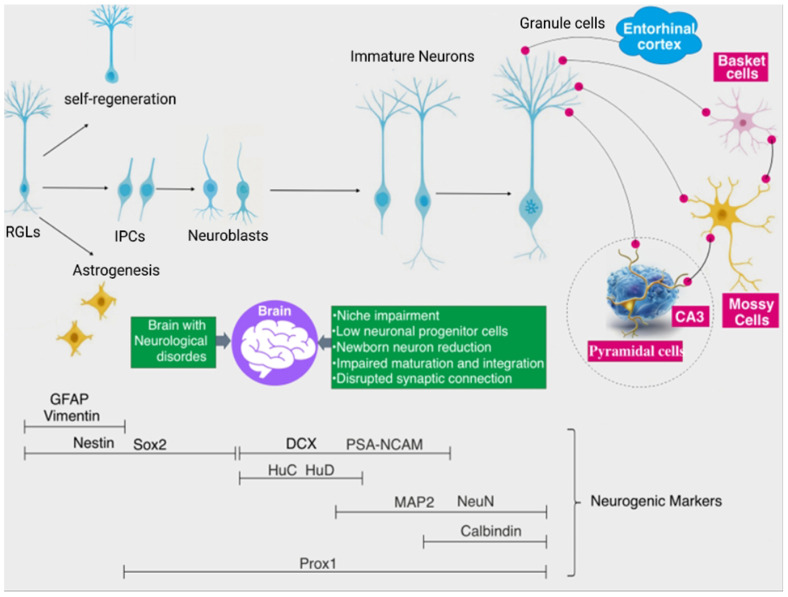
Proliferation, division, and integration of adult hippocampal neurogenesis (AHN) in mammals. The hippocampal dentate gyrus (DG) contains a specialized subgranular zone where radial glia-like neural stem cells (RGLs) reside. These RGLs are activated by signals from the neurogenic niche to embark on distinct developmental trajectories: (i) symmetric self-renewal to maintain the RGL pool; (ii) neurogenesis; (iii) asymmetric division into one RGL and one non-RGL; (iv) symmetric division into two non-RGLs; or (v) astrogenesis. Activation triggers profound genomic and metabolic shifts, including the upregulation of the achaete-scute homologue (ASCL1) and cell-activation-related metabolism. In rodents, newborn cells transition through sequential stages marked by specific neurogenic proteins—such as Sox2 (stemness), DCX (immature neurons), and NeuN (mature neurons)—ultimately developing into excitatory, glutamatergic granule neurons. However, this process is highly competitive; many cells perish within three weeks of division, with fewer than 25% surviving to integrate into functional circuits. This fine-tuned AHN process is frequently disrupted in neurodegenerative disorders like AD, leading to impaired proliferation, reduced differentiation, dysregulated maturation/integration, and compromised synaptic connectivity, all of which contribute to cognitive dysfunction. SGL, Subgranular layer; IPCs, Intermediate progenitor cells; GFAP, Glial fibrillary acidic protein; DCX, Doublecortin; NeuN, Neuronal nuclei; Prox1, Prospero homeobox protein 1; PSA-NCAM, Polysialylated-neural cell adhesion molecule; MAP2, Microtubule-associated protein 2.

Residing at the apex of the neurogenic hierarchy, hippocampal neural stem cells (NSCs) constitute a unique, self-renewing reservoir that serves as the fountainhead of nascent neurons [43,44] (Figure 1). These progenitors are sequestered within a specialized niche, where their quiescent-to-active transitions and lineage commitments are orchestrated by a sophisticated interplay between local cellular constituents, the extracellular matrix, and various cortical and subcortical projections [45] (Figure 1). NSCs are mostly in a dormant, quiescent state [46,47,48]. Upon stimulation by niche-derived signals—such as injury, stress, learning, or physical exercise—quiescent NSCs can become activated and undergo fate decisions, including self-renewal to maintain the NSC pool, differentiation into new neurons, or generation of other neural cell types [49] (Figure 1). Newly generated immature neurons typically require several months to fully mature. During this prolonged developmental period, they must compete with pre-existing neurons for synaptic integration into established neural circuits [50,51], resulting in the elimination of a substantial fraction of newborn neurons after division [52,53]. As maturation proceeds, immature neurons form mossy fiber synapses with pyramidal neurons in the CA3 region [50,54]. Synaptic connections among the entorhinal cortex (EC), dentate gyrus (DG), and CA3 are dynamically modified in response to experiential demands, thereby facilitating the resolution of memory interference. Newborn neurons play a critical role in this process by enhancing hippocampus-dependent memory functions, including memory discrimination, consolidation, and clearance [20,55,56]. The resolution of memory interference involves a process known as pattern separation (PS) [57,58,59,60,61,62,63]. Newborn neurons contribute to accelerating the consolidation of prior experiences and enhancing the precision of long-term memories [55,64,65]. Interestingly, the continuous incorporation of new neurons also drives “active forgetting”, which degrades or clears existing information stored in hippocampal circuits and then provides substrates for new learning [21,66]. The sequential stages of newborn neuron development are characterized by the expression of distinct neurogenic markers, including SRY-box transcription factor 2 (Sox2), doublecortin (DCX), and neuronal nuclei (NeuN)) [67,68]. However, this fine-tuned process of AHN is frequently derailed in neurodegenerative conditions like AD. Such pathologies manifest as aberrant proliferation, stunted differentiation, and compromised synaptic connectivity, ultimately precipitating cognitive decline [29,69]. Together, AHN is a vital process for maintaining mammalian brain plasticity and cognitive function by enabling the continuous generation and integration of new neurons into hippocampal networks in response to environmental changes and experiential demands.

### 3.2. Mapping AHN in Mammals with scRNA-Seq

The rapid expansion of snRNA-seq, supported by standardized laboratory protocols and advanced computational frameworks, has transformed neuroscience research and enabled the construction of comprehensive cellular and molecular brain atlases [70,71,72,73]. scRNA-seq allows high-precision classification of cellular subpopulations based on transcriptomic profiles, surpassing the limitations of traditional molecular markers and facilitating the identification of rare or previously uncharacterized cell types. For example, scRNA-seq has been applied to systematically map the cellular architecture of the mammalian cerebral cortex, revealing 47 transcriptionally distinct subpopulations. These clusters showed strong concordance with established cell identities defined by morphology, anatomical location, and canonical marker expression [70]. Beyond cell-type cataloguing, scRNA-seq provides fine-grained insights into the relationships between specific molecular signatures and disease-associated cellular states. In the *murine* striatum, this approach delineated ten major cell types and, notably, resolved two previously underappreciated subtypes of medium spiny neurons [74]. Each subtype displayed distinct gene expression patterns, including markers such as *Pcdh8*, *Tacr1*, and *Elavl4*. Dysregulation of these genes has been linked to neuropsychiatric disorders [74], including cognitive dysfunction and addiction. Now, scRNA-seq has been widely utilized to classify and characterize cells from diverse brain regions, and profiling signature genes of cell types in the brain offers insights into their functionality and relationship to health and disease.

Early scRNA-seq studies of DG cells primarily focused on identifying NSCs and progenitor developmental stages, defining key molecular determinants, and characterizing the molecular and cellular components of neurogenic niche pools. By applying a GluR1^−^/Cd24^−^ double-negative selection strategy, hippocampal niche cells were subclustered, revealing that neural stem cells and neuronal progenitors comprise heterogeneous populations occupying distinct developmental stages [33]. Early-stage NSCs expressed genes associated with stem cell quiescence (e.g., *Clu*, *Apoe*, and *Id3*), whereas late-stage NSCs expressed genes linked to activation (e.g., Fgfr3) [33]. Additionally, neural progenitors represent a distinct subtype, expressing cell-type-specific genes that are largely distinct from NSC-specific genes. Neural progenitors can be grouped into multiple subpopulations—namely, early, intermediate, and late progenitors—each defined by divergent gene expression patterns. How adult NSC pools are established and maintained remains incompletely understood. The integration of scRNA-seq with lineage-tracing approaches has enabled detailed characterization of transitions between developing, dormant, and activated NSCs in the rodent forebrain. The shift from highly active embryonic radial precursors (ERPs) to quiescent NSCs preserves substantial molecular and phenotypic similarity to the original ERP state [75]. Upon reactivation in adulthood to generate neurons, NSCs reacquire an ERP-like transcriptional identity. These findings suggest that transitions between ERPs and NSCs may not entail fundamental changes in cell identity but rather reflect dynamic interconversion between activated and quiescent NSC states. Taken together, scRNA-seq technology allows the exploration of the diversity and complexity of the brain at a single-cell level, enabling the identification of novel cell clusters and subclusters based on their gene expression patterns, as well as tracing the developmental trajectories and regulating mechanisms of NPCs.

### 3.3. Profiling AHN in Non-Human Primates with scRNA-Seq

Using diverse approaches—including BrdU labelling and immunohistochemistry—a substantial body of evidence has established the presence of AHN in the DG of non-human primates (NHPs) [76,77,78,79,80,81]. However, this neurogenesis proceeds at a slower pace and exhibits a more prolonged maturation period than that observed in rodents [76,77,78,79,80,81]. The extended developmental timeline may partly explain why AHN was not detected in earlier studies employing ^3^H-thymidine labeling [82], as this technique primarily marks cells actively undergoing DNA replication. More recently, scRNA-seq analyses have provided compelling additional evidence for the presence of AHN in non-human primates [22,23,26,83]. Analysis of postmortem hippocampal tissue from *cynomolgus macaques* across different ages identified a population of NPCs in the adult dentate gyrus and showed that ageing alters the expression of genes involved in adult neurogenesis and synaptic plasticity [83]. Complementary single-nucleus RNA sequencing (snRNA-seq) studies further mapped the molecular and cellular dynamics of the *macaque* hippocampus, confirming the presence of NPCs and immature neurons in the adult DG and revealing a previously unrecognized astrocyte-like population that may contribute to the regulation of AHN [26]. Recent studies have delineated multiple NPC and immature neuron subtypes in the NHP hippocampus, each characterized by distinct molecular signatures [22,23]. These findings suggest that scRNA-seq and snRNA-seq technologies have substantially refined our understanding of adult neurogenesis in NHPs, illuminating its existence, regulatory architecture, and potential roles in both physiological and pathological contexts. Moreover, accumulating evidence indicates that AHN in NHPs may exhibit distinct functional characteristics and regulatory mechanisms compared with other mammals, particularly rodents.

Collectively, AHN represents a fundamental mechanism underlying mammalian brain plasticity, providing a continuous supply of newborn neurons that integrate into existing hippocampal circuits to modulate learning and memory in response to environmental stimuli. The advent of scRNA-seq has revolutionized this field by enabling a granular, single-cell exploration of the rodent brain, effectively mapping previously hidden cell clusters, lineage-specific developmental trajectories, and the intricate regulatory networks governing neural progenitor cells. These technological advances have extended to non-human primate research, confirming the persistence of AHN across species while uncovering primate-specific regulatory features that diverge from rodent models. Despite these insights, profiling the single-cell landscapes of the postmortem *human* brain remains a challenge, hampered by rapid mRNA degradation and the technical difficulty of capturing the transcriptomic signatures of neurogenic precursors in aged or preserved *human* tissue.

## 4. snRNA-Seq Reshapes AHN Research in *Humans*

### 4.1. Debates on Human AHN

The continuous generation of new neurons in the hippocampus enhances brain plasticity, thereby supporting higher-order cognitive functions such as problem-solving and memory consolidation [31,67,84]. These capacities are essential for *human* adaptation and success in complex social environments, particularly those requiring advanced cognitive abilities. Consequently, considerable attention has been directed toward determining the extent to which AHN persists in the human hippocampus and the functional benefits it may confer. Eriksson et al. [85] conducted pioneering work demonstrating the existence of adult neurogenesis in the *human* hippocampus by incorporating the thymidine analogue bromodeoxyuridine (BrdU) into the DNA of dividing cells. Those BrdU-positive cells co-expressed simultaneously neuronal markers, hence supporting the existence of newly generated granule cells in the adult *human* hippocampus. Subsequent studies expanded upon these findings, further supporting the presence of adult neurogenesis in *humans* [6,86,87,88,89,90,91,92]. Among those studies, one research successfully isolated and cultured neural progenitor cells from postmortem hippocampal samples, which might provide methodologically independent evidence on *human* AHN [90].

Although evidence supporting the existence of AHN in *humans* continues to accumulate, discrepancies among reported findings persist [24,93]. For example, Sorrells et al. [24] reported that although new neurons are continuously generated in the DG of many adult mammals, the number of proliferating progenitors and immature neurons declines sharply after birth and is rarely detected in adulthood. Such inconsistencies may stem from methodological differences, the vulnerability of postmortem *human* brain tissue, variability in sample processing, and the lack of stable and sensitive markers specific to newborn neurons [5,7,8,9,94]. While Eriksson et al. [85] and Spalding et al. [95] provided evidence for adult neurogenesis in *humans* using more sophisticated techniques that detected the incorporation of BrdU and ^14^C into dividing granule cells, respectively, Sorrells et al. [24] based their conclusions solely on IHC-derived data. Furthermore, two studies [9,96] examined how different fixation methods and durations influence the detection of DCX^+^ cells in postmortem *human* brain samples. They demonstrated that prolonged fixation (e.g., several months) in commercial formalin—a common practice in many brain banks—substantially reduces the detectability of DCX^+^ cells within the same individuals. These observations suggest that tissue vulnerability during processing may contribute significantly to inconsistent reports of adult hippocampal neurogenesis in *humans*. Moreover, immunohistochemical analyses of postmortem *human* tissue typically rely on a narrow set of markers, such as DCX and PSA-NCAM, which may further constrain the detection of *human* adult neurogenesis [7,8]. Another possible explanation for the inconsistent findings is the relative scarcity of dividing cell populations and immature adult-born neurons in the adult *human* hippocampus compared with rodents, making these cells difficult to detect using traditional approaches [97]. Therefore, the application of more advanced methodologies, such as scRNA-seq and snRNA-seq, may help overcome these limitations by characterizing cell populations based on comprehensive transcriptomic signatures rather than a small number of pre-selected markers.

### 4.2. snRNA-Seq Reveals Novel Evidence on AHN in the Human Brain

While scRNA-seq effectively maps non-human mammalian brain taxonomy using fresh or flash-frozen tissue, it struggles with archived *human* autopsy samples. Because the protocol requires high-fidelity single-cell suspensions, the freezing and fixation typical of archived tissue often compromise results [98,99]. In addition, the reliance of scRNA-seq on polyadenylated RNA can bias transcript measurements, and the isolation process itself can alter the transcriptomic profiles of large, complex cells like neurons [100,101,102]. In contrast, snRNA-seq shows priority in profiling gene expression patterns in cells difficult to isolate or of archived tissues. Nucleus isolation for snRNA-seq analysis can be handled easily and quickly with frozen or preserved tissues, not significantly affecting the transcriptomic features of a cell. Generally, snRNA-seq can overcome several limitations associated with scRNA-seq and protein-based marker approaches that hinder the detection and characterization of adult-born neurons in *human* brain specimens. Current evidence has demonstrated that snRNA-seq and scRNA-seq often have a similar performance in detecting specific cell types, but snRNA-seq displays certain advantages over scRNA-seq, such as decreasing the effects of cell dissociation and cell size on cellular population enrichment, reducing transcriptional changes due to cellular stress, and capturing nascent mRNA for temporal studies [103,104]. Nonetheless, the divergences between whole-cell versus nucleus RNA sequencing, which are largely derived from technical issues linked with sampling and recovery of divergent subsets of the transcriptome, can partially account for the limitation of snRNA-seq in distinguishing some important proliferating molecules, such as Ki67, in NPCs [1,26,105]. To date, multiple snRNA-seq studies have identified NSCs, intermediate progenitors, neuroblasts, and immature neurons in the adult *human* hippocampus (Table 1), and have further characterized their transcriptional dynamics and disease relevance [1,26,106].

### 4.3. snRNA-Seq Can Discover Neuronal Progenitors in the Human Hippocampus

snRNA-seq provides a powerful strategy for overcoming the technical challenges associated with profiling postmortem brain tissue and often uncovers findings that are difficult to capture using traditional approaches. Using an improved high-throughput platform, one study applied massively parallel snRNA-seq with Drop-seq to profile single-nucleus transcriptomes from archived *human* brain tissue [108] (Table 1). This strategy generated high-quality transcriptomic data from preserved adult postmortem hippocampal samples and identified a cluster of 201 hippocampal cells classified as NSCs based on established marker gene expression [108]. It is important to note that this pioneering study was primarily designed to characterize the overall cellular and molecular diversity of the hippocampus, rather than to specifically define NSCs, progenitor developmental stages, or the molecular and cellular components of neurogenic niche pools. Interestingly, the NSC cluster from this dataset was subsequently reanalyzed by Sorrells and colleagues, who reported enrichment for ependymal cell markers [8]. This finding underscores the importance of applying diverse computational strategies to validate cell-type clustering results. Subsequently, Ayhan et al. [106] profiled nuclei isolated from surgically resected hippocampal specimens obtained from epilepsy patients to further characterize the cellular and molecular complexity of the *human* hippocampus. They identified seven clusters (Gra.Neu1–Gra.Neu7) exhibiting dentate gyrus granule cell identity, among which the Gra.Neu5 cluster uniquely expressed the recently described neuronal stem cell marker *LPAR1* [123]. Remarkably, another single-cell transcriptomic study on *murine* DG identified *Lpar1^+^* RGLs in the adult *murine* hippocampus [124]. Based on these findings, the authors proposed that the population of cells in Gra.Neu5 potentially represented precursor cells with neurogenic capacity. However, since this study did not conduct further analysis to define the transcriptome features of this specific subcluster and to determine the trajectories of cell-developing states, the exact nature of this subpopulation remains unclear. One notable limitation of broadly profiling hippocampal cellular diversity without developing targeted approaches to define NSC-specific genes and niche-associated molecular determinants is the potential omission of rare cell populations and subtle distinctions between closely related cell types, such as astrocytes and radial glia-like cells. This issue is exemplified by a recent study in which only astrocytes were reported, without further delineation of astrocytic subpopulations [17]. Nevertheless, the application of snRNA-seq continues to advance our understanding of AHN at single-nucleus resolution

### 4.4. snRNA-Seq Mapping Neurogenic Trajectories in the Human Hippocampus

Shortly after the publication of the aforementioned snRNA-seq study on *human* AHN [108], two additional snRNA-seq investigations further refined the characterization of cell populations, transcriptomic markers, and neurogenic trajectories in the adult *human* hippocampus [1,26] (Table 1). One study employed differentially expressed genes (DEGs) and cell-type–specific markers initially identified in *murine* models and subsequently validated in nonhuman primates to identify newborn neurons and reconstruct neurogenic trajectories in the aged *human* hippocampus [26]. A total of 22,119 nuclei from four postmortem *human* hippocampal samples (aged 67–92 years) were profiled by snRNA-seq, resulting in the identification of 13 distinct clusters, including a cluster of immature neurons [26]. Most NSCs were found to reside in a quiescent state, with only a small fraction exhibiting activated characteristics and expressing active NSC markers such as *ASCL1* and *HES6*. Lineage trajectory inference positioned the immature neuron cluster between the NSC and granule cell clusters and further resolved it into two subtypes: a neuroblast-like cluster and a late immature granule cell cluster [26].

However, snRNA-seq studies of AHN have not uniformly yielded positive evidence for adult neurogenesis. For example, Franjic et al. [23] identified only a single intermediate neuronal progenitor-like cell and one putative neuroblast among hippocampal granule cells, representing merely 0.003% of the profiled population. Several studies have proposed that methodological differences may partially account for these discrepancies. Wang et al. [26] directly addressed this issue by integrating their own snRNA-seq dataset with the Franjic et al. [23] dataset using the Canonical Correlation Analysis algorithm. The integrated analysis recovered 12 distinct cell types, with strong correspondence across all major cell populations except for the immature neuron cluster. They revealed that this immature neuron cluster was positioned adjacent to the integrated granule cell populations expressing neuroblast markers that were not detected in Franjic’s dataset. Wang et al. further revealed that astrocytes provide signals to mediate the dynastic states of adult NSCs [26]. Thus, they suggested that adult-born neurons existed and that AHN was persistent in older people.

Given the limitations of conventional unsupervised approaches in detecting immature granule cell populations within *human* snRNA-seq datasets, Zhou and colleagues [1] developed an enhanced analytical framework that integrates machine learning–based methods to profile gene expression at single-nucleus resolution. This powerful approach was subsequently applied in *human* postnatal hippocampal samples across child, adolescent, adult, and ageing stages. Cells exhibiting high similarity to the *human* immature granule cell signature were identified in hippocampal specimens across all age groups. The proportion of immature granule cells among total granule cells ranged from 9.4% in infancy to 3.1–7.5% beyond four years of age, closely aligning with estimates derived from DCX immunohistochemical analyses [93,96]. Importantly, they applied this model to reanalyze several previously published snRNA-seq datasets [23,106,108] and confirmed the presence of immature granule cells in all three snRNA-seq datasets, especially including the one originally reporting an extremely low occurrence rate of adult neurogenesis [23]. Therefore, this validated machine learning–based analytical framework proved to be a novel, sensitive, and specific strategy for identifying *human* immature granule cells across multiple brain snRNA-seq datasets and effectively addressed a major gap in our knowledge about adult *human* neurogenesis.

In summary, while accumulating evidence supports the presence of AHN in *humans*, inconsistencies across studies underscore the need for more refined methodologies. snRNA-seq has emerged as a powerful alternative to scRNA-seq, particularly for analyzing archived or postmortem *human* brain tissue where mRNA integrity is compromised. By enabling transcriptome-wide profiling at single-nucleus resolution—without reliance on limited protein markers—snRNA-seq offers a more sensitive and specific approach to identifying immature granule cells and dissecting AHN-related cellular diversity and developmental trajectories. Recent advances, including machine learning–driven analytical pipelines, have further enhanced the detection of *human* adult-born neurons in snRNA-seq datasets, helping to bridge critical knowledge gaps. Nevertheless, existing datasets derive from neurologically healthy individuals, underscoring the urgent need to apply these tools to well-annotated patient cohorts across various neurodegenerative diseases and clinical stages to fully understand how AHN is modulated—or lost—in pathological contexts.

## 5. snRNA-Seq Profiles AHN in Neurological Disorders

### 5.1. snRNA-Seq Profiles AHN and Transcriptomic Features in Epilepsy

**Figure 2:**
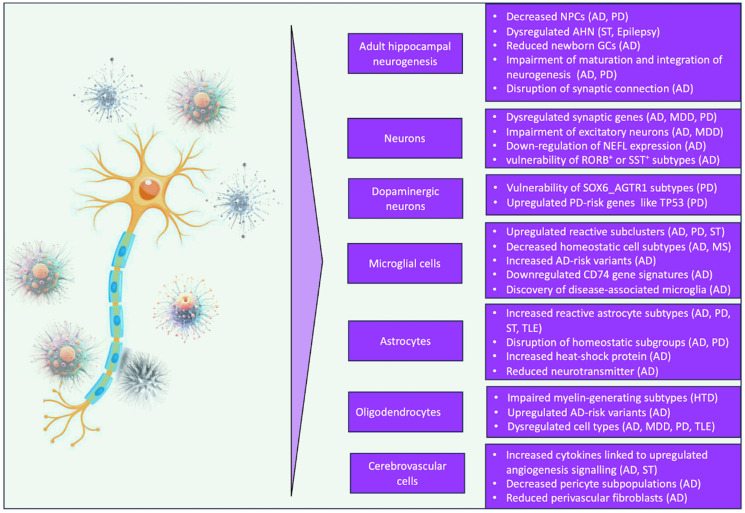
A schematic summary of the changes in cell types associated with neurological disorders. This figure summarizes widespread dysregulation across major brain cell types in neurological disorders. Key populations—including adult hippocampal neurogenesis, neurons, dopaminergic neurons, microglia, astrocytes, oligodendrocytes, and cerebrovascular cells—show distinct abnormalities across disorders such as Alzheimer’s disease (AD), Parkinson’s disease (PD), major depressive disorder (MDD), stroke (ST), epilepsy, multiple sclerosis (MS), temporal lobe epilepsy (TLE), and Huntington’s disease (HTD). Notable changes include reduced neural progenitor cells and impaired neurogenesis, synaptic and neuronal vulnerability, reactive microglial and astrocytic states, myelin-related deficits, and vascular dysfunction. The central neuron symbolizes the interconnected nature of these cellular disturbances within brain pathology.

The application of snRNA-seq to *human* hippocampal tissue has substantially advanced our ability to dissect the transcriptional dynamics and regulatory mechanisms that govern AHN under neuropathological conditions. Resolving cell states at single-nucleus resolution enables the tracing of disease-associated perturbations in lineage trajectories, regulatory networks, and the broader neurogenic niche (Figure 2). A notable example is provided by Ayhan et al. [109], who profiled surgically resected hippocampal specimens from epilepsy patients to characterize cell-type–specific transcriptional signatures related to learning and memory. Their analysis identified a distinct DG subcluster enriched for the neural stem cell marker *LPAR1*, previously recognized as a defining feature of RGL stem cells in the adult rodent hippocampus [124]. The presence of this *LPAR1*-positive population in *human* epileptic tissue underscores the conservation of neurogenic cell types across species, while also raising questions about how seizure activity may alter their functional potential.

**Figure 3:**
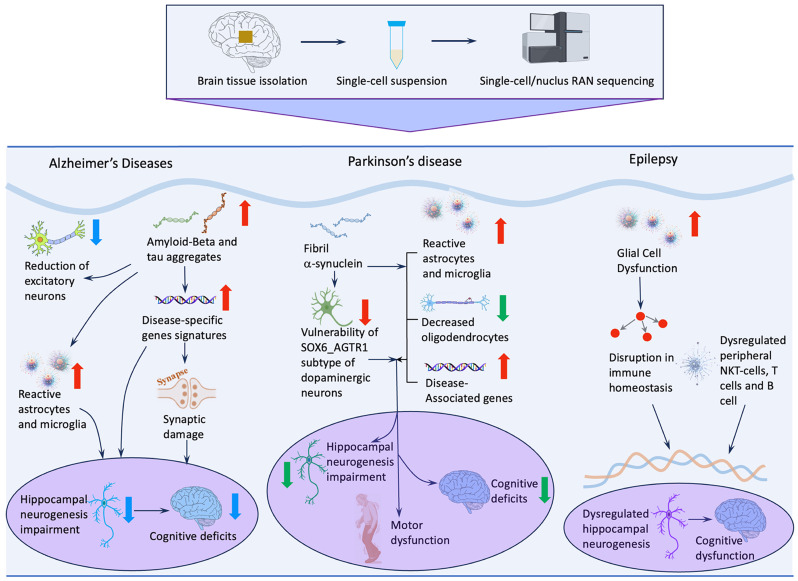
Disease-associated variations in cell populations and marker genes and subsequent impact on hippocampus neurogenesis and cognition in neurological diseases. High-resolution snRNA-seq analyses illuminate the diverse cellular states and risk-associated variants present in these conditions, revealing how disease perturbs neuronal and glial identities, transcriptional programs, and neurogenic pathways. By clarifying the mechanisms underlying impaired adult hippocampal neurogenesis (AHN) and cognitive decline, these datasets highlight precise cell-type targets for therapeutic strategies aimed at restoring neurogenic potential and hippocampal plasticity.

Epilepsy is increasingly recognized as a disorder in which neurogenic processes are disrupted not only by aberrant electrical activity but also by widespread transcriptomic alterations driven by inflammation and glial remodeling. snRNA-seq has been instrumental in elucidating these changes. For example, a distinct glial subpopulation has been identified in *human* neocortical tissue from individuals with temporal lobe epilepsy (TLE), characterized by a hybrid transcriptional profile co-expressing *GFAP* and *OLIG2*—markers typically associated with reactive astrocytes and oligodendrocyte precursor cells (OPCs), respectively. This hybrid phenotype suggests that chronic seizures may drive glial cells into atypical or transitional states, potentially reshaping neurogenic signaling and local circuit stability. Additional evidence for inflammation-driven transcriptomic remodeling comes from studies of refractory epilepsy, in which recurrent seizures activate key inflammatory pathways, including IL-1 and TLR signaling [125]. Cytokine–chemokine array analyses further demonstrate widespread inflammatory engagement in brain regions involved in autonomic and cardiorespiratory regulation. These findings enhance our understanding of how uncontrolled seizures contribute to systemic physiological risk and highlight inflammation-related pathways as potential therapeutic targets to reduce the incidence of sudden unexpected death in epilepsy [125]. Collectively, advances in snRNA-seq technologies have transformed our understanding of epilepsy by generating detailed cellular and molecular maps of disease-associated alterations (Figure 3). These approaches illuminate the mechanisms by which epilepsy perturbs AHN, reshapes glial and neuronal states, and contributes to cognitive dysfunction. By delineating the transcriptional programs that drive disease progression, single-cell technologies provide promising avenues for identifying therapeutic strategies aimed at restoring neurogenic capacity and mitigating neurological decline.

### 5.2. snRNA-Seq Reveals AHN Disruption and Transcriptomic Remodeling in AD

AD profoundly disrupts adult AHN and cognitive function, largely through the accumulation of misfolded Aβ and tau proteins. Yet, the transcriptional programs that underlie these neurogenic impairments have remained only partially understood. Recent advances in snRNA-seq have begun to fill this gap by providing unprecedented resolution of cell-type-specific vulnerabilities within the *human* hippocampus. A recent study applying snRNA-seq to hippocampal tissue from eight AD patients and thirteen controls demonstrated that immature granule cells (GCs) are particularly sensitive to AD pathology [1]. These cells were localized within defined GC clusters, and their abundance was reduced by approximately 50% in AD compared with age-matched controls. This observation aligns with immunohistochemical evidence showing diminished DCX-positive immature neurons in AD hippocampi [96,105]. Additionally, snRNA-seq identified fourteen down-regulated genes in immature neurons from AD patients, many of which are involved in synaptic plasticity, intracellular signaling, and activity-dependent remodeling [1]. These transcriptional deficits suggest that even surviving immature neurons may be functionally impaired, potentially limiting their integration into hippocampal circuits. Importantly, snRNA-seq datasets also illuminate how AHN changes across the *human* lifespan. The proportion of immature GCs is estimated at ~9.4% in infancy, but declines sharply to 3.1–7.5% from early childhood through adulthood [1]. Complementary work examining immature neuronal signatures across healthy, AD, and dementia-resilient individuals further refines this notion. Despite advanced age, the *human* hippocampus retains a population of immature neurons across all groups. Resilient individuals activate neuroprotective, anti-inflammatory, anti-amyloidogenic, and pro-myelination programs, suggesting an adaptive response that preserves cognitive function. AD patients, in contrast, exhibit a breakdown of these protective pathways, implying that dysfunctional—rather than absent—immature neurons may contribute to cognitive decline and loss of resilience [126].

Beyond neurogenesis, scRNA-seq has played a critical role in mapping the cellular transitions that accompany Alzheimer’s disease (AD) progression (Figure 2). A major contribution has been the characterization of dynamic microglial state changes. Single-cell profiling delineates a continuum from homeostatic microglia to disease-associated microglia (DAM), while also identifying additional states, including IFN-responsive, MHC-II–expressing, and proliferative microglia [127]. These subtypes represent distinct inflammatory and phagocytic programs that shape the neurodegenerative milieu and may indirectly influence AHN. snRNA-seq has also clarified neuronal vulnerability to tau pathology. Transcriptomic analyses of neurons containing neurofibrillary tangles (NFTs) revealed that at least 20 neocortical neuronal subtypes exhibit heightened susceptibility to NFT formation and degeneration [128]. NFT-bearing neurons display increased expression of genes associated with synaptic transmission and cellular stress responses, suggesting that tau pathology may provoke maladaptive attempts to sustain synaptic function. Collectively, snRNA-seq studies have demonstrated that genes regulating neurotransmitter release, synaptic vesicle recycling, and glutamate metabolism are consistently altered in neurons in AD [109]. These transcriptional disruptions provide mechanistic insight into how AD perturbs AHN and contributes to cognitive decline. By uncovering cell-type–specific vulnerabilities and disease-associated molecular pathways, single-cell technologies offer promising avenues for therapeutic strategies aimed at restoring neurogenic capacity and preserving cognitive resilience.

### 5.3. Application of snRNA-Seq in Other Neurological Disorders

Although PD is classically defined by the progressive degeneration of dopaminergic neurons in the substantia nigra, emerging evidence indicates that its pathological impact extends beyond the nigrostriatal system. Increasingly, PD is recognized as a disorder that also disrupts hippocampal circuitry and suppresses adult neurogenesis. A recent paired multi-omics study integrating snRNA-seq and snATAC-seq in the *human* midbrain provided a comprehensive view of age- and disease-associated transcriptional remodeling across glial and neuronal populations [118]. In healthy individuals, regulatory motifs linked to *NRF2* and *ASCL1*—factors associated with cellular resilience, oxidative stress responses, and neurogenic potential—were enriched within glial chromatin landscapes. These motifs were diminished in PD tissue, suggesting that the disease compromises not only neuronal integrity but also the supportive and regulatory functions of glial cells that contribute to brain homeostasis and neurogenic capacity. Although PD is classically defined by the degeneration of dopaminergic neurons in the substantia nigra, the specific neuronal subtypes that are most vulnerable have remained unclear. High-resolution scRNA-seq has identified ten transcriptionally distinct dopaminergic neuron subpopulations in the *human* substantia nigra, yet only one subtype—*SOX6_AGTR1*—undergoes selective and pronounced degeneration in PD patients [121] (Figure 2). Notably, this vulnerable subtype exhibits strong enrichment for genes associated with PD genetic risk, including *TP53* and *NR2F2*, suggesting that intrinsic molecular features may predispose this population to degeneration [121]. This discovery provides a mechanistic framework for understanding selective neuronal vulnerability in PD. Beyond neuronal populations, snRNA-seq has revealed disease-associated transcriptional alterations in glial cells. Gene expression patterns in both glial and neuronal populations correlate with PD-risk variants, including elevated *LRRK2* expression in microglia and increased *SNCA* expression in dopaminergic neurons [119]. Moreover, microglia and astrocytes from idiopathic PD (IPD) brains exhibit upregulation of genes involved in cytokine signaling, unfolded protein responses, and cell-cycle activation, indicating heightened inflammatory and proteostatic stress responses [119]. These findings highlight the active contribution of glial dysfunction to PD pathogenesis and suggest that targeting glial-specific pathways may offer new therapeutic opportunities.

AHN is highly sensitive to a broad spectrum of pathological conditions, and single-cell transcriptomic technologies have begun to clarify how diverse disease processes reshape the neurogenic niche. In prion diseases—characterized by prolonged asymptomatic phases followed by rapid cognitive and functional decline—the cellular dynamics of hippocampal neurogenesis have remained poorly defined. A recent scRNA-seq study by Slota et al. [129] identified disease-associated transcriptional alterations in neuronal populations, including dysregulation of genes involved in synaptic signaling and axon guidance. Intriguingly, these molecular changes coincided with a reduction in mature neurons and a relative increase in immature neuronal populations, suggesting a potential neuro-compensatory response aimed at replenishing neurons lost during disease progression scRNA-seq has also provided important insight into how environmental neurotoxins disrupt AHN. Cadmium, a well-established neurotoxicant, markedly alters NSCs’ differentiation trajectories, biasing them toward astrocytic rather than neuronal fates [130]. Cadmium exposure further induces subtype-specific transcriptional responses and perturbs intercellular communication within the NSC niche, underscoring the vulnerability of neurogenic microenvironments to toxicant-induced signaling disruption [130]. Similarly, scRNA-seq analysis of zebrafish brains exposed to varying concentrations of manganese (Mn) revealed a biphasic effect: low Mn levels promoted neurogenesis by expanding neural progenitor populations and supporting their differentiation into neurons and oligodendrocytes, whereas high Mn concentrations suppressed neurogenesis and impaired neural function [131]. These results indicate that Mn exerts dose-dependent effects on AHN and modulates intercellular communication through distinct signaling pathways. Despite these advances, snRNA-seq datasets remain limited across many neurological conditions. Critical gaps persist in our understanding of how AHN is altered in disorders such as PD, in which neurogenesis is likely to influence cognitive and emotional outcomes [32]. Expanding snRNA-seq analyses across these disease contexts will be essential for constructing a comprehensive framework of neurogenic vulnerability and resilience, ultimately guiding the development of targeted strategies to restore or enhance hippocampal plasticity.

Taken together, advances in snRNA-seq have greatly expanded our understanding of how neurological disorders—including epilepsy, AD, and PD—affect AHN. By generating high-resolution cellular and molecular landscapes, these technologies delineate disease-specific alterations in neuronal and glial states, transcriptional programs, and neurogenic pathways that contribute to impaired AHN and cognitive decline. Such insights highlight new cell-type-specific therapeutic targets aimed at restoring neurogenic capacity and supporting hippocampal plasticity. Expanding snRNA-seq investigations across diverse patient populations will be essential for defining patterns of neurogenic vulnerability and resilience; however, several key questions regarding AHN and its role in neurological disease must be resolved before meaningful clinical translation can be achieved.

## 6. Application of snRNA-Seq in Clinical Research

The rapid advancement of scRNA-seq technology has substantially deepened our understanding of the cellular diversity underlying hippocampal neurogenesis and neurological disorders. By enabling transcriptomic profiling at single-cell resolution, this approach has reshaped our view of neuronal heterogeneity and developmental dynamics, uncovering previously unrecognized layers of complexity with important implications for brain health and disease. Despite these technological advances, significant challenges remain—particularly in translating molecular insights into clinically actionable knowledge. Fundamental questions regarding how alterations in hippocampal neurogenesis contribute to disease initiation, progression, and therapeutic response remain unresolved, underscoring the need for rigorously designed clinical studies. Bridging the gap between single-cell discoveries and patient-level outcomes will require the integration of scRNA-seq data with detailed clinical phenotyping, longitudinal cohort analyses, and biomarker development. Such efforts are crucial to determine whether specific neurogenic signatures can function as diagnostic tools, prognostic indicators, or therapeutic targets in neurological disorders.

### 6.1. Insights into Hippocampal Neurogenesis and Neurological Disorders

The integration of scRNA-seq with advanced computational methodologies has transformed our understanding of the cellular and molecular architecture of the brain. For example, Del-Aguila and colleagues [132] performed snRNA-seq on postmortem AD brain samples and identified diverse neuronal populations, including excitatory and inhibitory neurons, as well as major glial cell types such as astrocytes, oligodendrocytes, and microglia. They reported a reduced proportion of excitatory neurons in AD brains and identified a distinct subcluster of DAM consistently expressing signature genes including *EEF1A1*, *GLUL*, *KIAA1217*, *LDLRAD3*, and *SPP1*. In a similar way, Wang et al. [26] delineated 13 distinct cell clusters within *human* hippocampal samples, including a population of immature neurons. Analyses of snRNA-seq datasets derived from the hippocampi of AD patients further revealed a significant reduction in the proportion of immature granule cells compared with healthy controls [1]. These studies demonstrate the power of scRNA-seq in refining our understanding of cellular classifications, uncovering new cellular subsets, and exploring the connections between neurodegenerative disorders and the generation of new neurons in the hippocampus. However, the cellular and molecular alterations underlying hippocampal neurogenesis across the spectrum of neurological disorders remain incompletely characterized. For instance, it remains to be addressed how PD, AD, or HTD affect various aspects of hippocampal neurogenesis, such as NPCs, immature neurons, and the maturation and integration process of newborn neurons, as well as their changes in response to different disease stages. Given the essential role of AHN in the formation and consolidation of memory, addressing these issues may help alleviate disease symptoms and enhance the quality of life for patients.

### 6.2. Revealing Disease Mechanisms and Pathways

scRNA-seq provides a unique opportunity to elucidate disease mechanisms and pathways by examining gene expression changes at the single-cell level. By comparing transcriptomic profiles of cells from healthy and diseased tissues, researchers can identify disease-associated gene signatures and uncover novel regulatory networks. Luquez et al. [15] reported widespread transcriptional alterations across major cell types in the AD brain, revealing profound compositional and functional shifts within the cellular landscape. In AD, reactive microglial populations are expanded and exhibit reduced expression of homeostatic and cell–cell signaling genes, including *CX3CR1*, *P2RY12*, and *P2RY13*, alongside disruption of pathways involved in metal ion homeostasis [15]. Endothelial cells in the entorhinal cortex display upregulation of genes associated with cytokine signaling and immune activation, such as *HLA-E*, *MEF2C*, and *NFKBIA*, changes that may contribute to blood–brain barrier (BBB) dysfunction [109]. Astrocytes similarly exhibit AD-related transcriptional signatures, including stress-responsive heat-shock proteins (*CRYAB*, *HSPA1A*, *HSBP1*, *HSP90AA1*), genes implicated in neurodegeneration (*NEAT1*), synaptogenesis (*NRXN1*, *NRXN3*), and neurotransmitter regulation [103,110,112,115,117]. Moreover, Single-cell transcriptomic mapping has further shown that certain neuronal subtypes exhibit lineage-specific vulnerability, suggesting that disease-associated alterations reflect selective susceptibility rather than uniform degeneration [133]. Consistent with this view, AHN and synaptic plasticity appear particularly vulnerable in AD [26]. Similarly, Couturier et al. have demonstrated that scRNA-seq analysis is also helpful in analyzing other neurological diseases, such as glioblastoma, to reveal cellular heterogeneity and gene expression patterns associated with disease progression. Although substantial evidence implicates neuroinflammation in the suppression of AHN in disorders such as AD and PD [134,135,136], the precise cellular and molecular processes that govern the relationship between neurological disorders and AHN remain incompletely defined. Therefore, elucidating the cellular and molecular interplay between neurological disorders and AHN at single-cell resolution is essential. Building on these insights, the development of therapeutic strategies aimed at enhancing hippocampal neurogenesis and synaptic plasticity through the mitigation of neuropathology warrants further investigation.

### 6.3. Enhancing Diagnostic Accuracy and Targeting Therapy

The integration of scRNA-seq into clinical research holds considerable promise for improving the identification and characterization of AHN and neurological disorders. Wang et al. [26] utilized snRNA-seq data to identify *ETNPPL* as a primate-specific neural stem cell marker and to validate *STMN1* and *STMN2* as markers of immature neurons in primates, which were subsequently confirmed in *human* brain tissue for detecting immature granule cells. By resolving disease-associated gene signatures at single-cell resolution, scRNA-seq provides a powerful framework for the precise classification and molecular stratification of neurological diseases. Castro et al. deconstructed the cellular architecture of pediatric brain tumors, unveiling novel subpopulations that offer a nuanced understanding of tumor heterogeneity and therapeutic vulnerabilities. By isolating specific cellular subclusters associated with disease progression or treatment resistance, scRNA-seq provides a robust framework for refining clinical prognostication. Similarly, Wang et al. [137] utilized scRNA-seq to examine glioblastoma specimens and pinpointed specific tumor cell subsets correlated with patient prognosis. Their research highlighted the predictive significance of cell diversity within glioblastoma and its capacity to direct individualized therapy methods. By pinpointing distinct cellular lineages or subpopulations that drive disease progression, researchers can engineer therapeutic interventions designed to modulate aberrant gene expression patterns characteristic of specific pathologies. For instance, Liu et al. [138] applied scRNA-seq to identify cell types linked to amyotrophic lateral sclerosis, leading to the discovery of novel therapeutic targets for this disease.

In summary, although scRNA-seq has driven substantial progress in identifying novel therapeutic targets, several critical questions remain. These include the validation of disease-associated cellular and molecular alterations and the determination of their functional and therapeutic relevance across diverse neurological models. A particularly active area of investigation focuses on elucidating how biological variation in AHN relates to pathological burdens—such as tau and α-synuclein accumulation—with the goal of preserving neurogenic capacity and cognitive function. Given the complexity of transcriptomic remodeling in neurological disorders, continued research will likely uncover additional gene signatures and disease-specific cellular subpopulations with translational potential.

## 7. Conclusions

The adult rodent brain retains the capacity to generate new neurons in the dentate gyrus (DG), a process essential for maintaining cerebral plasticity and cognitive function. However, whether comparable neurogenesis persists in the adult *human* hippocampus remains controversial and incompletely defined. Recent advances in scRNA-seq have provided unprecedented insight into the transcriptional architecture and cellular composition of AHN in postmortem *human* brain tissue. Furthermore, AHN is adversely affected by neurological disorders such as AD and PD, conditions that are closely linked to cognitive decline. To better understand the interplay between AHN and neurological disorders, scRNA-seq offers a powerful framework for both mechanistic investigation and clinical translation. scRNA-seq has become a powerful platform for advancing our understanding of neurological disorders by providing high-resolution insight into cellular diversity and gene expression dynamics. Its application across a broad range of neurological diseases has enabled the identification of novel cellular subpopulations, disease-associated gene signatures, and candidate molecular targets. Building on these advances, scRNA-seq shows strong potential for integration into clinical research, enabling more precise disease stratification and therapeutic development in neurology. Moreover, by elucidating cellular complexity and molecular mechanisms underlying these disorders, scRNA-seq provides a critical framework for developing targeted therapeutic strategies and ultimately improving patient outcomes.

Despite substantial progress, the cellular and molecular alterations underlying hippocampal neurogenesis across diverse neurological conditions remain incompletely characterized, warranting further investigation. The integration of emerging snRNA-seq datasets provides a promising strategy to resolve longstanding debates by establishing more rigorous conceptual and analytical frameworks. First, a working model of *human* adult hippocampal neurogenesis (AHN) derived from snRNA-seq data could generate a comprehensive transcriptional roadmap, clarify the molecular logic governing each stage of differentiation, and serve as a reference framework for identifying pathological deviations Second, distinguishing genuine adult neurogenesis from prolonged neuronal immaturity—by resolving single-cell transcriptional heterogeneity—would enable clearer delineation of these states across multiple dimensions, including transcriptional profiles, developmental trajectories, functional properties, and regulatory mechanisms. Third, targeted experimental and computational strategies are required to address current controversies, many of which arise from methodological limitations. Integrating snRNA-seq with rigorous experimental validation and advanced analytical pipelines may provide definitive solutions to these challenges. Overall, the convergence of standardized experimental protocols and state-of-the-art snRNA-seq–based analytical approaches holds substantial promise for defining the core features and regulatory principles of *human* AHN. Such advances will lay a critical foundation for elucidating its clinical relevance in neurodegenerative diseases, psychiatric disorders, and related neurological conditions.

## Figures and Tables

**Table 1:** Representative studies analyzing cellular and molecular characteristics of hippocampal neurogenesis and neurological disorders with single-cell transcriptomics.

Studies	Donors or Samples (*n*)	Regions of Interest	Cells/Nuclei (*n*)	Analyzing Methods	Changes in Cell Types/Subtypes and Disease-Associated Genes
AHN					
Dumitru, I. et al. [107]	25 subjects in the range of 0 to 78 years old	Hippocampus	115,861	snRNA-seq	Identification of proliferating neural progenitors
Ayhan, F. et al., 2021 [106]	5 adult people	Hippocampus	129,908	snRNA-seq	Presence of a specific subcluster of granule cells expressing stem cell marker–*LPAR1*
Franjic, D. et al., 2022 [23]	2 females and 4 males	Hippocampus	139,187	snRNA-seq	Detection of one cell with characteristics of intermediate neuronal progenitors and one putative neuroblast
Habib, N. et al., 2017 [108]	5 adult people	Hippocampus	14,137	snRNA-seq	Identification of 201 NPCs
Wang, W. et al., 2022 [26]	4 adult people	Hippocampus	22,119	snRNA-seq	Obvious presence of AHN with big between-individual variances
Zhou, Y. et al., 2022 [1]	72 males and females	Hippocampus	152,184	snRNA-seq	Immature GCs are about 3.1–7.5% of DG in adults, with twofold lower in AD patients
AD					
Grubman, A. et al., 2019 [109]	12 AD patients and normal controls	Entorhinal cortex	13,214	snRNA-seq	Up-regulated AD-risk genes like *LINGO1* in subclusters of oligodendrocytes, OPCs, astrocytes, and microglia
Zhou, Y. et al., 2020 [110]	21 AD patients and 11 controls	Prefrontal cortex Dorsolateral prefrontal cortexes	73,419 66,311	snRNA-seq	AD mice: Increased *Trem2*-dependent DAM and *Serpina3n^+^C4b^+^* reactive oligodendrocytes AD patients: Increased *IRF8*-driven reactive microglia Enhanced axonal myelination-impaired and metabolic adaptative oligodendrocytes Metabolic- dysregulated astrocytes
Cain, A. et al., 2023 [111]	24 individuals with AD	Dorsolateral prefrontal cortex	172,659	snRNA-seq	Increased AD-related neuronal and glial cells *Tau*-specific subtypes of oligodendrocytes, endothelial cells, astrocytes, and microglia
Lau, S.F. al., 2020 [112]	12 AD patients and 9 normal controls	Dorsolateral prefrontal cortex	169,496	snRNA-seq	Fewer neuroprotective astrocytes and oligodendrocytes A subtype of AECs expressing AGF and their receptors such as *EGFL7*, *FLT1*, and *VWF*
Olah, M. et al., 2020 [113]	10 AD, 4 MCI and 3 TLE	Dorsolateral prefrontal cortex, temporal cortex	16,242	scRNA-seq	Reduction in one distinct microglia subcluster in AD brain
Leng, K. et al., 2021 [103]	10 AD patients	Superior frontal gyrus, entorhinal cortex	106,136	snRNA-seq	Expression of the *RORB* gene in vulnerable excitatory neurons Down-regulated homeostatic function genes in a reactive astrocyte subpopulation
Gerrits, E. et al., 2021 [114]	20 AD patients and 9 age-matched controls	Occipital cortex, occipitotemporal cortex	482,472	snRNA-seq	The abundance of two distinct microglial subtypes associated with *Aβ* and *tau* phosphorylation
Morabito, S. et al., 2021 [115]	23 AD patients and 15 controls	Prefrontal cortex	191,890	snRNA-seq, snATC-seq	DAM and astrocytes expressing AD-relevant transcription factors like *SREBF1* Identification of two specific genes (*SPI1* and *NRF1*) linked to late-stage AD
Yang, A.C. et al., 2022 [116]	9 AD patients and 8 controls	Superior frontal cortex, Hippocampus	143,793	snRNA-seq	Selective vulnerability of ECM-maintaining pericytes with gene expression patterns involving dysregulated blood flow
Sadick, J.S. et al., 2022 [117]	9 AD patients and 5 controls	Prefrontal cortex	65,180	snRNA-seq	Disease-associated astrocytes dysregulating genes critical for neuronal plasticity Dysfunction of oligodendrocytes
PD					
Adams, L. et al., 2024 [118]	10 young and 9 older people, 15 PD patients	Brain	69,289	snRNA-seq	Motifs for *NRF2* and *ASCL1* decreased
Smajic, S. et al., 2022 [119]	6 IPD patients and 5 matched controls	Midbrain	41,000	snRNA-seq	Presence of IPD-specific microglia and astrocytes Microglia show a specific pro-inflammatory trajectory *ECADPS2* is specifically expressed in dysfunctional dopaminergic neurons
Wang, P. et al., 2022 [120]	8 PD patients and 6 age-matched healthy controls	Blood	10,466	scRNA0seq	Increased memory B cells and decreased naïve B cells Enhanced antigen presentation capacity of B cells
Kamath, T. et al., 2022 [121]	10 PD patients and 8 matched controls	Midbrain	387,483	snRNA-seq	A specific subset of dopaminergic neurons susceptible to PD pathology is defined by the expression of *AGTR1*.
Epilepsy					
Sarkis, R. et al., 2023 [122]	87 epilepsy subjects and 20 healthy controls	Peripheral blood	84,000	scRNA-seq	Increased proportion of memory *CD4^+^* and *CD8^+^* T-cells and NK T-cells expressing cytotoxic cytokines such as granzyme and perforin in poorly controlled epilepsy subjects Reduction in *CD14^+^* and *CD16^+^* monocytes, and B memory cells

Notes: AHN, Adult hippocampal neurogenesis; DAM, disease-associated microglia; OPCs, Oligodendrocyte precursor cells; NPCs: neuronal progenitor cells; scRNA-seq, Single-cell RNA sequencing; snRNA-seq: Single-nucleus RNA sequencing; PD, Parkinson’s disease; AD, Alzheimer’s disease; GCs, Granule cells; AECs: angiogenic endothelial cells; AGF: angiogenic growth factors; ECM, extracellular matrix; IPD: Idiopathic Parkinson’s Disease; NK, natural killer; MCI, Medical College of Indiana; TLE, Technology and Livelihood Education.

## Data Availability

No new data were generated or analyzed in this study.

## Abbreviations

AHN	Adult Hippocampal Neurogenesis
scRNA-seq	Single-cell RNA sequencing
snRNA-seq	Single-nucleus RNA sequencing
AD	Alzheimer’s Disease
PD	Parkinson’s Disease
NSCs	Neural Stem Cells
IHC	Immunohistochemistry
SGZ	Subgranular Zone
PS	Pattern Separation
DEGs	Differentially Expressed Genes
ERPs	Embryonic Radial Precursors
NHPs	Non-Human Primates
BrdU	Bromodeoxyuridine
DCX	Doublecortin
PSA-NCAM	Polysialylated Neural Cell Adhesion Molecule
ASCL1	Achaete-Scute Family BHLH Transcription Factor 1
HES6	Hairy and Enhancer of Split 6
LPAR1	Lysophosphatidic Acid Receptor 1
RGLs	Radial Glial Cells
MDD	Major Depressive Disorder
MS	Multiple Sclerosis
NFTs	Neurofibrillary Tangles
OPCs	Oligodendrocyte Precursor Cells
Mn	Manganese
IPD	Idiopathic Parkinson’s Disease
LRRK2	Leucine-Rich Repeat Kinase 2
SNCA	Alpha-Synuclein
DAM	Disease-Associated Microglia
BAG3	BCL2-Associated Athanogene 3
GBM	Glioblastoma Multiforme
SULT1E1	Sulfotransferase Family 1E Member 1
MO	Meningioma Organoid
NKT	Natural Killer T Cells
LE	Temporal Lobe Epilepsy
GFAP	Glial Fibrillary Acidic Protein
OLIG2	Oligodendrocyte Transcription Factor 2
PBMCs	Peripheral Blood Mononuclear Cells
PC	Poorly Controlled
WC	Well-Controlled
IL-1	Interleukin-1
TLR	Toll-Like Receptor
XRCC6	X-Ray Repair Cross-Complementing Protein 6
Csf1	Colony-Stimulating Factor 1
Ccl6	Chemokine (C-C motif) Ligand 6
Trem2	Triggering Receptor Expressed on Myeloid Cells 2
Tyrobp	TYRO Protein Tyrosine Kinase Binding Protein
Clec7a	C-Type Lectin Domain Family 7 Member A
APP/PS1	Amyloid Precursor Protein/Presenilin 1
PSEN1-E280A	Presenilin 1 E280A Mutation
APOE3	Apolipoprotein E3
Cplx1	Complexin-1
iPSC	Induced Pluripotent Stem Cell
IGSF3	Immunoglobulin Superfamily Member 3
EEF1A1	Eukaryotic Translation Elongation Factor 1 Alpha 1
GLUL	Glutamine Synthetase
KIAA1217	Protein KIAA1217
LDLRAD3	Low-Density Lipoprotein Receptor Adaptor Protein 3
CX3CR1	CX3C Chemokine Receptor 1
P2RY12	Purinergic Receptor P2Y12
P2RY13	Purinergic Receptor P2Y13
HLA-E	Human Leukocyte Antigen-E
MEF2C	Myocyte Enhancer Factor 2C
NFKBIA	NF-Kappa-B Inhibitor Alpha
CRYAB	Crystallin Alpha B
HSP1A1	Heat Shock Protein Family A Member 1
HSBP1	Heat Shock Factor Binding Protein 1
HSP90AA1	Heat Shock Protein 90 Alpha Family Class A Member 1
NEAT1	Nuclear-Enriched Abundant Transcript 1
NRXN1	Neurexin 1
NRXN3	Neurexin 3
ETNPPL	Ethanolamine Phosphotransferase 1-Like Protein
STMN1	Stathmin 1
STMN2	Stathmin 2
